# Evaluation of Bayesian alphabet and GBLUP based on different marker density for genomic prediction in Alpine Merino sheep

**DOI:** 10.1093/g3journal/jkab206

**Published:** 2021-06-26

**Authors:** Shaohua Zhu, Tingting Guo, Chao Yuan, Jianbin Liu, Jianye Li, Mei Han, Hongchang Zhao, Yi Wu, Weibo Sun, Xijun Wang, Tianxiang Wang, Jigang Liu, Christian Keambou Tiambo, Yaojing Yue, Bohui Yang

**Affiliations:** 1 Animal Science Department, Lanzhou Institute of Husbandry and Pharmaceutical Sciences, Chinese Academy of Agricultural Sciences, Lanzhou 730050, China; 2 Sheep Breeding Engineering Technology Center, Chinese Academy of Agricultural Sciences, Lanzhou 730050, China; 3 Gansu Provincial Sheep Breeding Technology Extension Station, Sunan 734400, China; 4 Centre for Tropical Livestock Genetics and Health (CTLGH), International Livestock Research Institute, Nairobi 00100, Kenya

**Keywords:** genomic prediction, Alpine Merino sheep, wool traits, GBLUP, Bayesian alphabet, marker density, GenPred, shared data resource

## Abstract

The marker density, the heritability level of trait and the statistical models adopted are critical to the accuracy of genomic prediction (GP) or selection (GS). If the potential of GP is to be fully utilized to optimize the effect of breeding and selection, in addition to incorporating the above factors into simulated data for analysis, it is essential to incorporate these factors into real data for understanding their impact on GP accuracy, more clearly and intuitively. Herein, we studied the GP of six wool traits of sheep by two different models, including Bayesian Alphabet (BayesA, BayesB, BayesCπ, and Bayesian LASSO) and genomic best linear unbiased prediction (GBLUP). We adopted fivefold cross-validation to perform the accuracy evaluation based on the genotyping data of Alpine Merino sheep (*n* = 821). The main aim was to study the influence and interaction of different models and marker densities on GP accuracy. The GP accuracy of the six traits was found to be between 0.28 and 0.60, as demonstrated by the cross-validation results. We showed that the accuracy of GP could be improved by increasing the marker density, which is closely related to the model adopted and the heritability level of the trait. Moreover, based on two different marker densities, it was derived that the prediction effect of GBLUP model for traits with low heritability was better; while with the increase of heritability level, the advantage of Bayesian Alphabet would be more obvious, therefore, different models of GP are appropriate in different traits. These findings indicated the significance of applying appropriate models for GP which would assist in further exploring the optimization of GP.

## Introduction

The advancement in the field of quantitative genetics and molecular biology has improved the selection and breeding methods of domestic animals (Rabier *et al.* 2016). Meuwissen *et al.* (2001) proposed a more advantageous selection method, known as genomic selection (GS) or genomic prediction (GP; Meuwissen *et al.* 2001). This method combines the genome-wide single nucleotide polymorphism (SNP) with phenotypic data and implicates them for genetic evaluation (Goertzel *et al.* 2006; Iwata and Jannink 2011; Su *et al.* 2012; Taylor *et al.* 2016). It was first applied to the dairy cows (Taylor *et al.* 2016) and is now widely used in other model animals such as beef cattle (Taylor *et al.* 2012), pigs (Cleveland and Hickey 2013), goats (Carillier-Jacquin *et al.* 2018), and sheep (Werf 2009), aquatic animals like Atlantic salmon (Tsai *et al.* 2015), rainbow trout (Vallejo *et al.* 2016), and plants (Legarra *et al.* 2008; Desta and Ortiz 2014), such as wheat (Poland *et al.* 2012) and alfalfa (Jia *et al.* 2018). GS has made a substantial contribution to the modern breeding process, as compared with traditional methods; the main advantages of this method include improved estimation accuracy of breeding value (BV; Sun *et al.* 2014; Weller *et al.* 2017), increased genetic progress, and reduced breeding costs (Miglior *et al.* 2017; Wiggans *et al.* 2017). With the successive publication of various livestock genome sequences and the continuous upgrade of commercial SNP microarrays, different types and densities of microarrays have been adopted in the GP of different livestock (Singh *et al.* 2019). Accuracy and cost are generally the most critical factors in GP, compared with low-density SNP microarrays, the high-density SNP microarrays could accommodate more SNP sites that may lead to higher coverage of the genotype data (Di *et al.* 2005). However, the cost of the high-density microarray was comparatively higher. In contrast, although the low-density SNP microarrays have fewer SNP sites, it is more applicable in population breeding with a huge dataset due to its lower cost. Both the methods have their own pros and cons and therefore, it is difficult to conclude which density microarray is best suitable for GP.

For the first time, Meuwissen *et al.* (2001) proposed a GS based on Bayes method, which includes BayesA and BayesB (Meuwissen *et al.* 2001). Based upon this approach, several other methods were also derived such as BayesCπ method (Habier 2011), Bayesian least absolute shrinkage and selection operator (Bayesian LASSO) method (Park and Casella 2008). Subsequently, Gianola (2013) summarized these methods as the Bayesian Alphabet method. In fact, the assumptions and strategies adopted by these methods are different. The BayesA assumes that all SNPs have genetic effects and the variance of marker effects should obey the t-distribution, whereas BayesB assumes that only a small proportion of SNPs have an effect. Furthermore, the BayesCπ is similar to BayesB, and estimates the proportion of sites with no effect of π in the model. The Bayesian LASSO method assumes that all markers have effects, and the variance of marker effects obeys the double exponential distribution also known as Laplace distribution (Gianola 2013). VanRaden (2008), proposed another calculation method for GP and named it as genomic best linear unbiased prediction (GBLUP). It calculates the relationship matrix of individuals via genome-wide genotype information instead of traditional pedigree information. Herein, the matrix denoted as G is applied to replace the A matrix in BLUP, to estimate the BVs according to the BLUP method (VanRaden 2008). Another novel approach known as single-step GBLUP (SSGBLUP or HBLUP) has been developed based on GBLUP (Aguilar *et al.* 2010). This method integrates the phenotype, pedigree and genomic information into a model, and combines the traditional kinship matrix A with the genome relationship matrix G according to different weights to construct a new relationship matrix H, then simultaneously estimate the genetic effects of all individuals (including individuals with and without genotypes). Although there are various GP methods available, no method could be suitable for all traits. Therefore, in this study, two methods based on Bayes and GBLUP models were adopted to study the prediction accuracy of real data for different wool traits, aiming to screen ideal GP models.

As an important domestic animal, sheep is one of the earliest domestic animals reared by humans (Wang *et al.* 2014) and provides diverse resources such as mutton, wool, skin, and milk. Merino and Merino-derived sheep breeds are distributed globally (Ciani *et al.* 2015). As the object of this study, the Alpine Merino sheep has Australian Merino and Tibetan sheep lineage. Thanks to their adaptation in high-altitude hypoxia and excellent wool quality, they quickly adapted to the freezing Qinghai-Tibet Plateau, living in high altitude and cold conditions for generations (Zhu *et al.* 2020). The length and strength of the staple and fiber diameter (FD) are closely related to the wool quality and are the important economic traits of fine-wool sheep. Therefore, adopting genome analysis to explore wool traits is crucial for the selection and development of this population. However, the application of GP in the Alpine Merino sheep population is still at the initial stage. According to the genomic information obtained by SNP microarray, combined with the phenotypic dataset closely related to wool traits, different methods can be used to conduct GP research and comparing the results, including the genetic effects of GP markers and GP methods for research. This has made an important contribution to the application of GP in the Alpine Merino sheep population.

In this study, two different densities of SNPs including low (50 K) and high (630 K) were applied to estimate the genetic variance components of the Alpine Merino sheep datasets. Further, based upon the SNP genotypes data, different models were adopted for GP and cross-validated to compare the accuracy of different GP methods. The main purpose of this study is to investigate the impact of different densities of SNP genotypes and different GP methods (Bayesian Alphabet and GBLUP) on the accuracy and optimization methods of GP in Alpine Merino sheep populations.

## Materials and methods

### Ethics statement

All animal work carried out in this study was performed per the guidelines for the care and use of laboratory animals promulgated by the State Council of the People’s Republic of China. The study was approved (License Number: 2019-008) by the Animal Management and Ethics Committee of Lanzhou, Institute of Animal Husbandry and Veterinary Sciences, Chinese Academy of Agricultural Sciences.

### Animal resources and phenotypic data

The original phenotypic dataset was obtained from the Sheep Breeding Technology Extension Station of Gansu Province. These datasets consisted of 11,500 individuals based on 7 different herds with information such as region (herd), sex, and date of birth. The individuals in this study included 821 Alpine Merino sheep (563 ewes and 258 rams) from HuangCheng pasture in Gansu Province, China, the pasture was under the jurisdiction of the Gansu Sheep Breeding Technology Extension Station which has a rigorously standardized system of breeding and management, to ensure that all the individuals have uniform feeding and management conditions. The average age of each individual with phenotypic data was about 12–14 months. The wool traits involved in this study were staple length (SL), clean fleece weight rate (CFWR), average FD, coefficient of variation of average FD (FD_CV), staple strength (SS), and fleece extension rate (FER). The wool from individuals was collected and evaluated according to the Agricultural Industry Standards of the People’s Republic of China (NO. NY/T 1236-2006). Wool samples (∼250–300 g) collected from the abdomen of each individual, were weighed and stored in ziplock bags (Xingdeli Packaging Material Company Ltd., Shenzhen, China). Within one week, the samples were sent to the National Animal and Rural Ministry of Animal and Fur Quality Supervision and Inspection Center (Lanzhou, China) for weighing, screening, and quality identification of wool. Blood samples (∼5 ml) were also collected from each sheep from the jugular vein and immediately transferred to the vacutainer blood collection tube (Yuli Medical Equipment Company Ltd., Jiangsu Province, China). Blood samples were stored at −20°C for further genotyping (Ma *et al.* 2019). The statistics used to estimate variance components and GP of each wool trait are presented in Table 1.

**Table 1:** Descriptive statistics of phenotypic values of traits

Trait	Abbreviation	SE	Mean ± SD	Numbers
Clean fleece weight rate (%)	CFWR	0.25	63.58 ± 7.10	817
Staple strength (N/ktex)	SS	0.28	33.81 ± 7.98	813
Fleece extension rate (%)	FER	0.18	19.67 ± 5.07	811
Mean fiber diameter (mm)	FD	0.07	20.81 ± 2.11	811
Coefficient of variation of FD	FD_CV	0.11	20.22 ± 3.26	816
Staple length (mm)	SL	0.46	90.63 ± 13.16	812

SE, standard error; SD, standard deviation.

### Genotypic data and population structure assessment

The customized Affymetrix HD 630K microarray was employed as the datasets for the genotype of high-density SNP genotypes (H-datasets) for the Alpine Merino sheep. The genotyping platform for analysis was based on the array plate processing workflow of GeneTitan system (Santa Clara, CA, USA) from Thermo Fisher (Affymetrix). The sites in the Illumina Ovine SNP 50K microarray were screened out from the Affymetrix HD 630K microarray and used as the datasets of low-density SNP genotypes (L-datasets). The H- and L-datasets were preprocessed using PLINK v1.9b4 software prior to the statistical analysis and variance component estimation (Purcell *et al.* 2007). The SNPs were eliminated with call rate (geno) below 95%, minor allele frequency (MAF) below 0.01, which seriously deviated from the Hardy Weinberg Equilibrium with a *P*-value below 10E-6. Here, the X, Y chromosomes and mitochondrial markers were excluded from the analysis. Beagle software (version number; 12Jul19.0df) was used to impute the sporadic missing alleles (Browing and Browing 2009; Wang *et al.* 2019). After quality control and imputation, a total of 821 individuals with 460,656 autosomal SNPs were retained for H-datasets, and 35,379 autosomal SNPs for L-datasets. In addition, based on the genotypic data, we adopted TASSEL 5.2.43 software (Bradbury *et al.* 2007) to perform PCA analysis on all the individuals involved in the study, then constructed and drew the principal component analysis plot.

### Statistical methods for GP

We explored the application of SNP datasets of different densities in genome evaluation and further compared the accuracy of GP adopting 5 different models, including Bayesian Alphabet (BayesA, BayesB, BayesCπ, and Bayesian LASSO) and GBLUP. Six wool traits from 821 samples were used to first, estimate the variance of each component, including the additive and residual variance; second, five different models were adopted to perform GP, and its accuracy was compared via fivefold cross-validation, and all these models were evaluated in SNP datasets of H- and L-datasets. Replicate measurements were not available for the individuals so that the effects of permanent environmental were not modeled. The samples involved were from different herds and sex. These factors altered the phenotype in a fixed pattern, and hence the system environmental effects were added to the framework.

The statistical methods of Bayesian Alphabet involved can be written as:

(1) $$y=Xb+\sum_{j}^{n}Z_{ij}\alpha_{j}+e$$

Here, y represents the corrected phenotypic value of individuals, Xb refers to a fixed term, and b contains a vector of three effects, including herds, sex, and mean of population. $Z_{ij}$ represents the genotype of individual i at site j, and $\alpha_{j}$ represents the effect value of site j, and therefore $\sum_{j}^{n}Z_{ij}\alpha_{j}$ refers to the BV corresponding to individual i, e to the vector of residual effects. According to the method from Meuwissen *et al.* and Habier *et al* (Meuwissen *et al.* 2001; Habier 2011), we adopted the R package “BGLR” to estimate the effect of markers (Pérez and de los Campos 2014). The hypothetical distribution of all markers' effects in different Bayes methods and the formula of effect distribution are shown in Table 2.

**Table 2:** Different GS methods and effects distribution in this study

Method	Assumed distribution of effect	Formula of effect distribution
GBLUP	Normal	$\beta_{i}$ ∼ $N(0,\sigma_{j}^{2})$
BayesA	t	$\beta_{i}$ ∼ $t(0,{\nu ,\sigma }_{j}^{2})$
BayesB	Point-t	βi=0 with probability πβi ∼ χ-2ν,S with probability (1-π)
BayesCπ	t mixture	$\beta_{i}$ ∼ $\pi t\left(0,{\nu ,\sigma }_{j}^{2}\right)$ + $\left(1-\pi )\;t(0,{\nu ,0.01\sigma }_{j}^{2}\right)$
Bayesian LASSO	Double exponential or Laplace	$\beta_{i}$ ∼ DE(0,θ)

The methods of GBLUP involved in this study correspond to a linear model.

(2) y=Xb+Zu+e

In Bayesian Alphabet model, in equation (2), y, b, e, and X represent the same parameters as those defined in equation (1), u is the vector of individuals BV, Z is the design matrix corresponding to the BV. The covariance matrix of additive effects is represented by $\mathrm{Var}\left(u\right)=G\sigma_{a}^{2}$, where G is the matrix of relationships between individuals obtained from genomic information, calculated according to the approach of VanRaden (VanRaden 2008; equation 3) and also implemented through the R package “BGLR” (Pérez and de los Campos 2014).

(3) $$G=\frac{W_{a}W_{a}^{T}}{2\sum_{f=1}^{m}p_{f}(1-p_{f})},$$

where $W_{a}$ represented the matrix of additive genetic effect markers, with dimension of the number of individuals (n) by the number of loci (m), and $p_{f}$ is the MAF value of locus f.

### Accuracy of GP by K-fold cross-validation

Fivefold cross-validation was performed to compare the accuracy of different methods of GP. During K-fold cross validation, the population should be divided randomly (de los Campos *et al.* 2009). The datasets consisting of 821 individuals were divided into five approximately equally sized subgroups (each subgroup contained around 165 individuals). For fivefold cross-validation, four subgroups which retain the phenotype and genotype, were regarded as training population (reference population) to estimate the parameters. The remaining subgroup that is, candidate population was used to verify the samples, and correspondingly, the phenotype of this group of samples was set as missing (Not applicable, NA).

GP accuracy is represented by the Pearson Correlation Coefficient between GEBV and the corrected phenotypic value ($y^{*}$) (Waldmann 2019). It calculates the correlation between two continuous variables, and the result is between [−1,1], where $\mathrm{Cov}\left(\mathrm{GEBV},y^{*}\right)$ represents the covariance of GEBV and $y^{*}$, Var(GEBV) and $\mathrm{Var}\left(y^{*}\right)$ represent the variance of GEBV and corrected phenotypic value, respectively. The larger the value of Correlation Coefficient, the higher the accuracy of prediction.

(4) $$\mathrm{COR}\left(\mathrm{GEBV},\;y^{*}\right)=\;\frac{\mathrm{Cov}\left(\mathrm{GEBV},y^{*}\right)}{\sqrt{Var(\mathrm{GEBV})\times \mathrm{Var}\left(y^{*}\right)}}$$

According to the above mentioned five models, the cross-validation was performed based on two types of genotypic data (H- and L-datasets), with different densities and the BVs of the validation group (candidate population) were predicted. In addition, the above cross-validation was performed in triplicates in order to ensure the randomness of individuals in the validation group. Finally, the GP accuracy values were calculated for each validation, averaged, and then recorded as the final accuracy.

### Data availability

Genotype and phenotype data are available, they could be obtained at Figshare (https://figshare.com/projects/Evaluation_about_GP_for_821_AMS/112614). The script adopted in this study can be obtained on GitHub (https://github.com/gdlc/BGLR-R).

## Results

### Phenotypic statistics and genotypic characteristics

A total of six wool traits were collected and the descriptive statistics of individual wool phenotype data were presented in Table 1, including the abbreviation of each trait, the corresponding standard error (SE), the average value (represented by mean ± SD), and the number of individuals that were effectively recorded (Numbers). For the wool traits, the SD ranged from 2.11 (FD) to 13.16 (SL), and the SE ranged from 0.07 (FD) to 0.46 (SL). In addition, the structure of the population is drawn based on the top three eigenvectors using principal component 1 (PC1), 2 (PC2), and 3 (PC3), the PCA plot (Supplementary Figure S1) showed that only a few of individuals have population stratification, it suggested that population structure has good homogeneity.

### The polygenic heritability and the GP accuracy

Estimate the phenotypic variation and additive variation of the six wool traits based on the L- and H-datasets, and calculates the heritability (h^2^) of each trait based on the ratio of the additive variance to the total phenotypic variance (Va/Vp). For L-datasets, heritability ranged from 0.37 (FER) to 0.70 (SL); and for H-datasets, heritability ranged from 0.29 (FER) to 0.68 (SL). The estimated results of heritability (expressed as the proportion of additive variance in phenotypic variance) shown in Table 3, states that SL was the highest and the FER was the lowest irrespective of the L- or H-datasets. Moreover, the heritability estimated by L-datasets was slightly higher than that of H-datasets for these six wool traits.

**Table 3:** Estimates of additive and residual components of variance obtained under GBLUP methodology using BGLR package for different datasets

Traits	Dataset type	$\sigma_{a}^{2}\;($ **SE)**	$h^{2}\;($ **SE)^*a*^**	$\sigma_{e}^{2}\;($ **SE)**
CFWR	L-Datasets	26.47 (0.33)	0.56 (0.01)	20.77 (0.24)
	H-Datasets	23.04 (0.26)	0.46 (0.01)	27.06 (0.25)
SS	L-Datasets	28.64 (0.42)	0.46 (0.01)	33.46 (0.35)
	H-Datasets	23.20 (0.43)	0.35 (0.01)	42.53 (0.38)
FER	L-Datasets	9.04 (0.17)	0.37 (0.02)	16.75 (0.16)
	H-Datasets	7.57 (0.18)	0.29 (0.01)	18.77 (0.18)
FD	L-Datasets	1.91 (0.03)	0.45 (0.02)	2.26 (0.02)
	H-Datasets	2.04 (0.03)	0.44 (0.01)	2.46 (0.02)
FD_CV	L-Datasets	5.46 (0.06)	0.56 (0.01)	4.13 (0.05)
	H-Datasets	5.75 (0.08)	0.55 (0.01)	4.65 (0.06)
SL	L-Datasets	89.63 (0.72)	0.70 (0.01)	37.73 (0.55)
	H-Datasets	106.99 (0.86)	0.68 (0.01)	50.64 (0.76)

CFWR, clean fleece weight rate; SS, staple strength; FER, fleece extension rate; FD, mean FD; FD_CV, coefficient of variation of FD; SL, staple length

a Polygenic heritability, the proportion of the additive effect variance to the total phenotypic variance.

**Figure 1:**
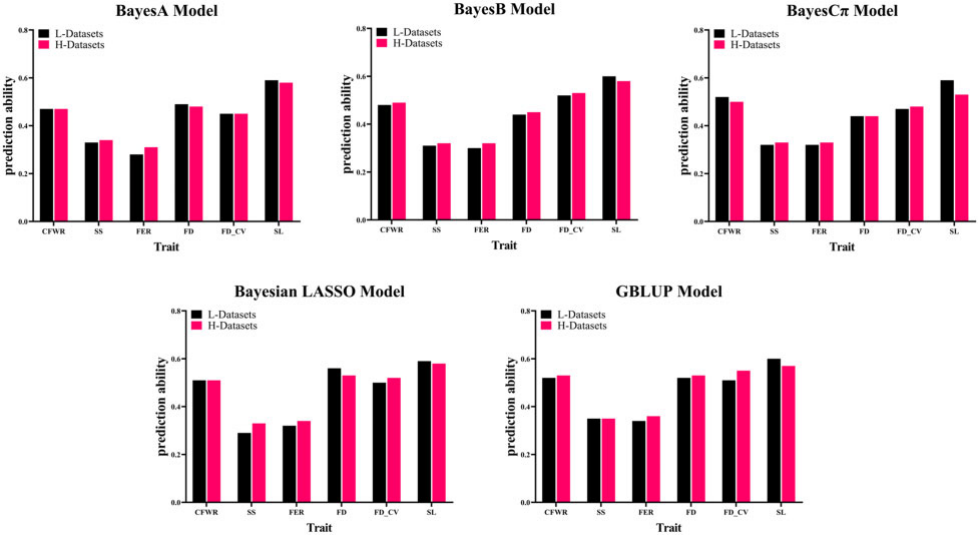
Comparison of GP accuracy based on different density genotype datasets. The six traits were CFWR, SS, FER, mean FD, FD_CV, and SL.

**Figure 2:**
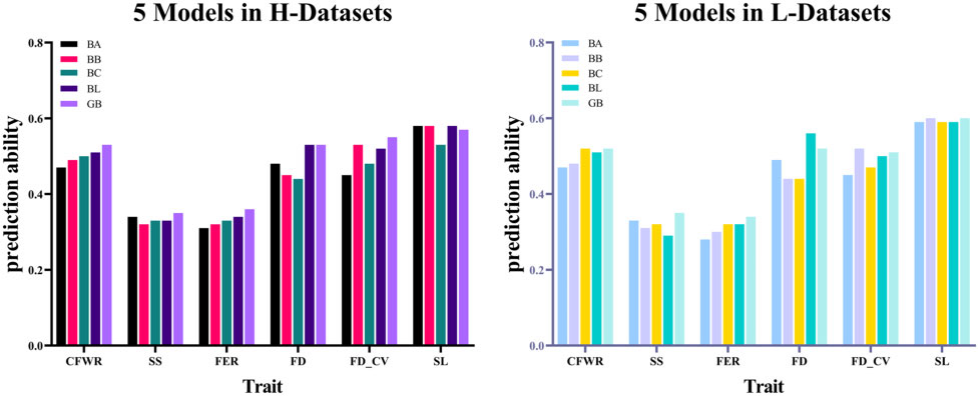
GP accuracy of five models in different heritability level. On the left is the result for the H-datasets, and on the right is the result for the L-datasets. The six traits were CFWR, SS, FER, mean FD, FD_CV, and SL. The five models were: BayesA (BA); BayesB (BB); BayesCπ (BC); Bayesian LASSO (BL); and GBLUP (GB).

The GP accuracy was calculated using five methods based on two marker density datasets (Table 4). For L-datasets, the GP accuracy of SL was the highest (0.59 for Bayesian LASSO model); and the GP accuracy of FER was the lowest (0.28 for BayesA model). Correspondingly, for H-datasets, the trait with the highest GP accuracy was also SL (0.58 for BayesA, BayesB, and Bayesian LASSO model), and the trait with the lowest GP accuracy was FER (0.31 for BayesA model; Figures 1 and 2) .

**Table 4:** Comparison of prediction accuracies of six traits based on two datasets via five models

Trait*^a^*		Prediction Accuracy*^b^*									
	Model*^c^*	BA		BB		BC		BL		GB	
	Dataset	L	H	L	H	L	H	L	H	L	H
CFWR		0.47 (0.01)	0.47 (0.03)	0.48 (0.02)	0.49 (0.02)	0.52 (0.01)	0.50 (0.03)	0.51 (0.02)	0.51 (0.03)	0.52 (0.01)	0.53 (0.02)
SS		0.33 (0.01)	0.34 (0.01)	0.31 (0.02)	0.32 (0.03)	0.32 (0.02)	0.33 (0.02)	0.29 (0.02)	0.33 (0.04)	0.35 (0.03)	0.35 (0.02)
FER		0.28 (0.01)	0.31 (0.03)	0.30 (0.03)	0.32 (0.02)	0.32 (0.01)	0.33 (0.03)	0.32 (0.01)	0.34 (0.02)	0.34 (0.01)	0.36 (0.01)
FD		0.49 (0.01)	0.48 (0.04)	0.44 (0.02)	0.45 (0.06)	0.44 (0.02)	0.44 (0.04)	0.56 (0.01)	0.53 (0.01)	0.52 (0.02)	0.53 (0.01)
FD_CV		0.45 (0.02)	0.45 (0.03)	0.52 (0.01)	0.53 (0.00)	0.47 (0.02)	0.48 (0.02)	0.50 (0.02)	0.52 (0.01)	0.51 (0.01)	0.55 (0.02)
SL		0.59 (0.02)	0.58 (0.01)	0.60 (0.01)	0.58 (0.01)	0.59 (0.01)	0.53 (0.02)	0.59 (0.02)	0.58 (0.02)	0.60 (0.03)	0.57 (0.02)

a Abbreviations of traits explained in Table 3

b SE are in parenthesis

c BA, BayesA; BB, BayesB; BC, BayesCπ; BL, Bayesian LASSO; GB, genomic best linear unbiased prediction, GBLUP.

## Discussion

### Genomic information and individual relationship matrix

The analyses involved in this study are all based on genomic information obtained from genotyping through microarrays, GP has replaced the traditional phenotype and pedigree information with the dense markers, providing a new method to estimate genetic variance, which improves the accuracy of prediction and selection (Daetwyler *et al.* 2012). Genomic information is not only suitable for a population with pedigree information, but can also be applied to populations without pedigree information or incorrect, incomplete and even missing genealogical records (Visscher *et al.* 2010; Yang *et al.* 2010), and this is also the main reason for adopting GBLUP model in this study. Due to the lack of pedigree information in the population involved in this study, in order to ensure the reliability of the estimation of individual relationship matrix, we have performed microarray genotyping for all individuals and constructed a G matrix, but did not adopted single‐step method (SS-BLUP) to construct H matrix (Guo *et al.* 2015), which will be more conducive to the subsequent heritability and GEBV estimation accuracy. In the GBLUP model, the traditional individual relationship matrix A constructed by pedigree was replaced by the genome matrix G, which represents the relationship between individuals more accurately, as it is based on a dense genome-wide markers. More importantly, this may capture the genetic connections from unknown common ancestors, because it represents confirmed gene sharing, and has advantages over presumed or conceptualized ancestral sharing (Su *et al.* 2012). In GBLUP model, it was assumed that each SNP has an effect, and the cumulative effect of SNPs obey a normal distribution (de los Campos *et al.* 2009), the assumption might only be applicable to certain specific groups or traits. According to the hypothesis of Habier *et al.* (2011), for some traits, only a few markers have a larger effect, while most markers have little or no effect (Liu *et al.* 2018). Therefore, GBLUP may not be suitable for such trait, in other words, the GP accuracy of GBLUP will be lower than other models, like the FD trait this study, the GP accuracy (0.56 based on L-datasets) of the Bayesian LASSO model was higher than that (0.52 based on L-datasets) of the GBLUP model. From the above results, GBLUP may not be applicable to FD traits and its predictive ability may not achieve satisfactory results. Hence, it is necessary to adopt different GP models. In the Bayesian Alphabet method, models such as BayesB and BayesCπ assume that most of the SNPs in the genome are located in regions without quantitative trait locus (QTL) and have no effect (Park and Casella 2008). whereas a small number of other SNPs existed in linkage disequilibrium (LD) together with QTL, and accounts for most of the effect (de los campos *et al.* 2009; Hayes *et al.* 2009). According to reports, different Bayesian Alphabet methods put forward a variety of prior hypotheses on the distribution of SNP effects (Table 2; de los campos *et al.* 2009). In this study, in addition to the GBLUP method, four typical Bayesian Alphabet methods (BayesA, BayesB, BayesCπ, and Bayesian LASSO) were also used to compare the GP accuracy of the six wool traits.

In most cases, GP suffers limitations while adopting the high- or low-density SNP genomic information, *i.e*., the number of marker effects that need to be estimated is often greater than the number of individuals to be recorded. In this study, both the L- and the H-datasets showed that the number (35,379 and 460,656) of markers was much larger than the number (821) of individuals. Although many advanced statistical methods (Erbe *et al.* 2012; Cheng *et al.* 2018) have been proposed to overcome this challenge, the true distribution of QTL and SNP effects were unclear for many quantitative traits (de Los Campos *et al.* 2009). Moreover, in contrast to L-datasets, the H-datasets microarrays contain more genomic information, but it also involves more complex matrices and larger computation, which will undoubtedly increase the cost of time and economy (Hayes *et al.* 2009).

### Phenotypic statistics and estimation of heritability

In this study, the collected phenotypic statistics of wool traits were compared with the results in previous reports: Moghaddar *et al.* collected 3000–8000 phenotypic records of various wool traits from different breeds of sheep in 2014, including the Poll Dorset, White Suffolk, and Border Leicester. In their report, the statistical mean values of FD and FD_CV were 19.93 ± 5.39 and 19.26 ± 2.86 (mean ± SD), respectively. The statistical mean of SS and SL was 33.82 ± 9.82, 80.93 ± 13.06, respectively (Moghaddar *et al.* 2014). In addition, according to the study by Hamadani *et al.* (2019) on Rambouillet sheep, where they collected and recorded the wool traits of 4108 samples from 1998 to 2007, the statistical mean value of FD and SL was 21.26 ± 0.03 (mean ± SE), 56.1 ± 0.05, respectively. The above comparison showed that the phenotypic statistics of this study were consistent with the earlier studies. It could be suggested that although the number of phenotypes collected in this study was not as large as their study (over 3000 individuals), the statistical values of phenotype measurement were still reliable.

The additive and residual variance, and the heritability of the six wool traits of the Alpine Merino sheep population were estimated, and we compared with previous studies in order to ensure the rationality of the estimation results. Daetwyler *et al.* (2010) and Moghaddar *et al.* (2014) conducted the genetic parameter estimation and GP studies based on pedigree information, the study involves multiple sheep breeds including Merino, Border Leicester, and White Suffolk. The results showed that estimated heritabilities of SS and SL were in the range from 0.37 to 0.55 and 0.56 to 0.67, respectively, and the estimated heritabilities of FD and FD_CV were between 0.62–0.75 and 0.47–0.57, respectively; Fogarty (1995) and Safari *et al.* (2005) collected and summarized the genetic parameters of nine wool traits. Their results showed that the estimated heritabilities of SS, SL, CFWR, FD, FD_CV were 0.34, 0.46–0.48, 0.34–0.51, 0.51–0.59, and 0.52, respectively; In addition, Bolormaa *et al.* (2017) conducted GP and genome-wide association study in Australian Merino sheep population based on SNP data. In their study, a total of 22 wool traits were collected, the estimation results of genetic parameters showed that the estimated heritabilities of SL and SS were 0.62 and 0.38, respectively; and the estimated heritabilities of FD and FD_CV were 0.84 and 0.60, respectively (Bolormaa *et al.* 2017). Moreover, according to the variance components estimation results of the H and L-datasets, the estimation results of FD and FD_CV were more consistent, and the results of other traits showed that the residual variance of the H-dataset was higher, it suggest that the high-density microarray data contained more sites, which brought more marker information and the number of QTLs (Ala Noshahr *et al.* 2017), leading to a more detailed division of genetic variance. However, in this study, except for the slightly lower estimated value of FD (0.42–0.47), the other four wool traits (Table 3) were close to the results reported in the previous literature, especially for the SS (0.33–0.46) and FD_CV (0.55–0.56) were very close to them. The comparison with the previous studies suggested that the heritability results estimated from the Alpine Merino dataset in this study were reliable.

### GP results and accuracy of prediction

In order for GS to be effectively applied to the breeding programs of livestock populations, it is necessary to fulfill a prediction study to deeply understand the factors that affect the prediction accuracy of the datasets before actual population selection, which is especially important for local breeds such as Alpine Merino sheep. Therefore, we collected 821 samples from the breeding program to investigate the influence and interaction of marker density and GP on the accuracy of prediction. Previous studies suggested that the density of markers has an essential impact on the accuracy of GP (Calus *et al.* 2008; Boustan *et al.* 2013). Solberg *et al*. (2008) adopted simulation to analyze the correlation between accuracy and marker density, their results showed that increasing the density of SNPs from 1 to 8/centimorgan (cM) could improve the accuracy of GP by 25% (Solberg *et al.* 2008), but this did not mean that the accuracy could always improve with the increase of marker density, in other words, there is a limit to this improvement. Heffner *et al.* (2011a, 2011b) conducted a study using a wheat dataset and showed that with the increased density from 192 to 1158 markers, the accuracy of GP could be improved by 10%. However, when the marker density increased from 192 to 384, it caused only a small increase in accuracy (Heffner *et al.* 2011a, 2011b). Most of the 10% improvement mentioned above occurred in the interval from 192 to 384 markers, and the increase of the remaining markers did not significantly affect the accuracy. These results indicate that marker density has a positive effect on the accuracy of GP, while the response of accuracy to density will eventually stabilize (de Los Campos *et al.* 2013).

Herein, we adopted the genome datasets based on the level of 50K and 630K microarray, respectively. Table 1 shows that with the marker density increases, the improved accuracy of GP for most traits, especially in SS and FER, model Bayesian LASSO and BayesA increased by 12% and 11%, respectively, whereas in other traits the accuracy was not significantly improved, such as CFWR and FD_CV, the accuracy of GBLUP and BayesB increased only by 1%; SS and FER benefited more from the increase in marker density than other traits, which could be explained by the fact that quantitative genetic characteristics require more markers to accurately estimate their many small effects of QTL (Zhang *et al.* 2015). Interestingly, there are exceptions in this study, for some traits, the accuracy may even decrease: in FD trait, the accuracy of BayesA and Bayesian LASSO models were reduced by 3% and 5%, respectively. Two reasons that may explain why increasing number of markers on each chromosome led to a decrease in GP accuracy. First, the number of markers in the microarray is much larger than the number of samples, which may be due to excessively high density of markers leading to the model overfitting (Heslot *et al.* 2012). Second, the increases in the number of markers will lead to the addition of more unknown variables (marker effects) and a lack of accurate estimation. The study from Fatemeh Ala Noshahr *et al.* (2017) also showed that with the number of SNPs increased from 2000 to 3000, both BayesA and GBLUP model indicated a decrease in the accuracy of GP. Our results suggest that increasing the density of markers could indeed improve the GP accuracy, but it is closely related to the trait itself. For traits with low heritability levels (FER and SS), a small part of the phenotypic variation was explained by additive effects (Medeiros *et al.* 2016), and the increase of marker density may improve the accuracy of GP more obviously; correspondingly, for those traits with high heritability levels (CFWR and FD), increasing the marker density has little benefit on the GP accuracy, sometimes it even has a negative impact on accuracy.

Among the six wool traits studied here, SL and FD_CV had the highest heritability (h^2^ = 0.53 and = 0.58, respectively), and their corresponding accuracy of GP was also the highest, which ranged from 0.53–0.60 to 0.45–0.55, respectively. While for two traits with the lowest heritability, SS (h^2^ = 0.33) and FER (h^2^ = 0.28), the accuracy was 0.29–0.38 and 0.28–0.36, respectively, which was lower than SL and FD_CV. For those traits with lower heritability, the correlation between phenotypic value and genetic value will be lower, the effect value of markers distributed across the genome may be estimated with lower accuracy (Habier 2011), it suggested that higher heritability has a positive effect on the accuracy of GP. Bolormaa *et al.* (2013) also reported that the prediction of the trait with the highest heritability was more accurate (Bolormaa *et al.* 2013), and also several studies have shown that the accuracy of GP increases with the improved heritability (Daetwyler *et al.* 2008, 2010), the results of this study agreed with them. In addition, we found that for traits with low heritability, GBLUP had a better prediction effect, whether it is adopting L- or H-datasets, but with the increase of heritability, the advantage of GBLUP is not obvious. From Table 4, it could be observed that for the trait SL with high heritability, the estimation accuracy of BayesB (0.58–0.60) and Bayesian LASSO (0.58–0.59) models performed better, this may indicate that for some traits with high heritability, BayesB and Bayesian LASSO assumes more reasonable distribution in marker effect, which leads to higher prediction accuracy. Similar results were obtained in the study of Honarvar and his coworkers, based on simulation data of three different levels of heritability, they compared the accuracy of the RRBLUP and Bayesian-LASSO models, and the results showed that the GP accuracy of the Bayesian-LASSO model is higher than that of the RRBLUP model for these traits, but the former has a more obvious advantage in traits with high heritability (Honarvar 2013), and it should be noted that GBLUP was equivalent to RRBLUP. In addition, the accuracy of GP was also related to the size and structure of the reference group (Heffner *et al.* 2011a, 2011b; Dreisigacker *et al.* 2014). We will collect and organize a larger dataset in future and try to take the above factors into consideration in subsequent studies for better conclusive results.

## Conclusions

To summarize, this study was based on two different densities of microarray genotyping data (50K and 630K), adopting Bayesian Alphabet (including BayesA, BayesB, BayesCπ, and Bayesian LASSO) and GBLUP model to perform the GP. The heritability of six wool traits of Alpine Merino sheep was estimated, and the accuracy of the BVs prediction of these traits under different conditions was evaluated through fivefold cross-validation. To the best of our knowledge, this was the first study of optimization of GP which has been applied to the domesticated Alpine Merino sheep populations. We have observed that for traits with low heritability (SS and FER), increasing the density of markers could improves the GP accuracy, but it has little impact on traits with high heritability (SL), and even decreases the accuracy (FD). The accuracy of the GBLUP model is generally higher than that of the Bayesian Alphabet model for SS and FER, while with the improvement of heritability, the advantage of GBLUP is no longer obvious. Therefore, from this study, we conclude that different GP models are applicable to different traits: GBLUP is more suitable for traits with lower heritability (FER and SS), and for Bayesian Alphabet, especially BayesB and Bayesian LASSO, have better GP effects for traits with high heritability (FD and SL). 
